# Recent advances in cancer fusion transcript detection

**DOI:** 10.1093/bib/bbac519

**Published:** 2022-12-16

**Authors:** Ryley Dorney, Bijay P Dhungel, John E J Rasko, Lionel Hebbard, Ulf Schmitz

**Affiliations:** epartment of Molecular & Cell Biology, College of Public Health, Medical & Vet Sciences, James Cook University, Douglas, QLD 4811, Australia; Centre for Tropical Bioinformatics and Molecular Biology, Australian Institute of Tropical Health and Medicine, James Cook University, Cairns 4878, Australia; Gene and Stem Cell Therapy Program Centenary Institute, The University of Sydney, Camperdown, NSW 2050, Australia; Faculty of Medicine & Health, The University of Sydney, Camperdown, NSW 2006, Australia; Centre for Tropical Bioinformatics and Molecular Biology, Australian Institute of Tropical Health and Medicine, James Cook University, Cairns 4878, Australia; Gene and Stem Cell Therapy Program Centenary Institute, The University of Sydney, Camperdown, NSW 2050, Australia; Faculty of Medicine & Health, The University of Sydney, Camperdown, NSW 2006, Australia; epartment of Molecular & Cell Biology, College of Public Health, Medical & Vet Sciences, James Cook University, Douglas, QLD 4811, Australia; Storr Liver Centre, Westmead Institute for Medical Research, Westmead Hospital and University of Sydney, Sydney, New South Wales, Australia; epartment of Molecular & Cell Biology, College of Public Health, Medical & Vet Sciences, James Cook University, Douglas, QLD 4811, Australia; Centre for Tropical Bioinformatics and Molecular Biology, Australian Institute of Tropical Health and Medicine, James Cook University, Cairns 4878, Australia

**Keywords:** fusion RNA, chimeric RNA, third-generation sequencing, trans-splicing, transcriptional readthrough

## Abstract

Extensive investigation of gene fusions in cancer has led to the discovery of novel biomarkers and therapeutic targets. To date, most studies have neglected chromosomal rearrangement-independent fusion transcripts and complex fusion structures such as double or triple-hop fusions, and fusion-circRNAs. In this review, we untangle fusion-related terminology and propose a classification system involving both gene and transcript fusions. We highlight the importance of RNA-level fusions and how long-read sequencing approaches can improve detection and characterization. Moreover, we discuss novel bioinformatic tools to identify fusions in long-read sequencing data and strategies to experimentally validate and functionally characterize fusion transcripts.

## Introduction

Fusion RNAs are RNA transcripts that contain exons and sometimes introns, from different parental genes. They are also referred to as fusion transcripts, chimeric transcripts, chimeric RNAs among other names. Traditionally, fusion RNAs are known to be produced by transcription of a gene fusion.

However, by our definition, fusion RNA refers to any hybrid transcript, reliant on gene annotation rather than mechanism of generation.

Gao *et al*. suggested that fusion events drive 16.5% of human cancers and function as the sole driver in more than 1% [1]. Fusions can contribute to oncogenicity by altering tumour suppressor or proto-oncogene expression. Alternatively, fusions may modify protein function by encoding a fusion protein and thereby stimulate tumorigenesis [2–4]. Some fusion proteins are immunogenic and give rise to neoantigens that can be targeted in personalized immunotherapy [5].

However, not all fusion RNAs are inherently oncogenic, as some have been observed in various healthy cells or tissues [6]. Fusion RNAs can contribute to cellular phenotypic plasticity by expanding genome functionality without increasing the number of genes and thus can provide modified cell survival mechanisms against environmental stress [7].

Previous literature reviews described fusion RNAs, their biosynthesis and occurrence in human cancers [8–12]. However, these surveys are of limited scope and do not extend to both recent advances in knowledge and methods for studying fusion RNAs. In this review, we untangle the terminology used in the context of gene and transcript fusions and discuss the recently observed multi-segmented fusions and circular RNA (circRNA) fusions. We describe the importance and function of fusion RNAs, their function as non-coding RNAs, and highlight the often overlooked RNA-level fusions. Additionally, we emphasize the opportunities that third-generation sequencing approaches hold for fusion RNA profiling and discuss the associated bioinformatics approaches and challenges. Finally, we discuss strategies for the complementary experimental validation of fusion RNAs.

## Terminology and classification of gene and transcript fusions

A clearly defined and universally accepted classification system and terminology for fusion RNAs are yet to be adopted. The fusion classification system proposed by Calabrese *et al*. separates genomic rearrangement-dependent fusions—caused by changes at the DNA-level—from genomic rearrangement-independent fusions—caused by changes at the RNA-level [13].

### Genomic rearrangement-dependent fusions

**Figure 1:**
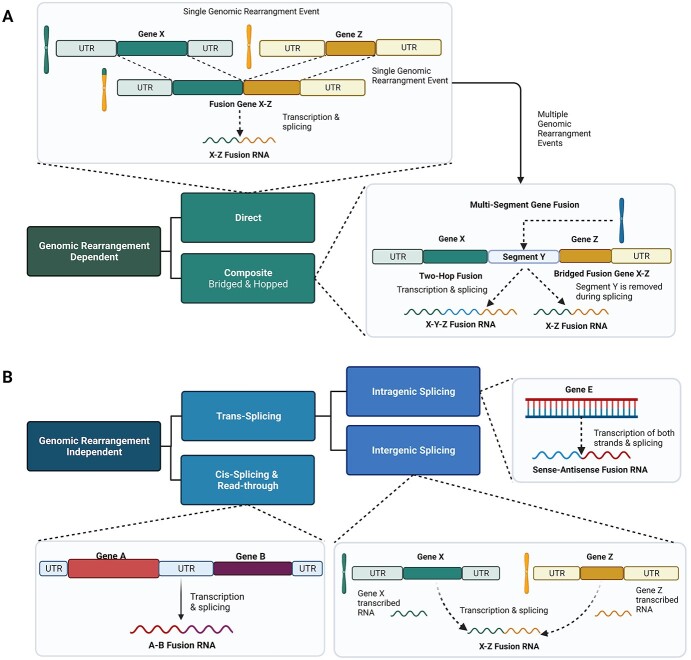
Classification scheme of fusion transcripts. The classification of fusion transcript origins and schematic examples of each fusion type are depicted. (**A**) Genomic rearrangement-dependent fusions are subdivided into direct and composite, depending on the number of rearrangement events that have occurred. Types of genomic rearrangement events that can act on fusions include deletions, inversions or translocations. (**B**) Genomic rearrangement-independent fusions are a result of distortions in alternative splicing or transcriptional readthrough mechanisms. UTR, untranslated region. Created with BioRender.com

Genomic rearrangement-dependent fusions are subdivided into direct (caused by a single structural rearrangement event) and composite fusions (a result of multiple structural rearrangements) (Figure 1A).

Composite fusions have an additional subclass known as bridged fusions, whereby a third genomic location bridges two genes, though this bridge section is often not retained in the mature RNA transcript [13]. It should be noted that bridged fusions are distinct from double-hop fusions, also referred to as 2-hop [14], wherein the internal genomic regions remain part of the mature RNA transcript [15]. These regions can be thousands of base pairs long, and for some double-hop fusions, splicing was observed in these regions [15]. Hopped fusions are not limited to only 2-hop fusions, as ‘hop’ refers to the number of structural rearrangement events involved in the fusion, and 3-hop fusions have also been observed [14]. Given that long-read sequencing is a relatively new technology, more complex RNA-level fusion events could be discovered.

### Genomic rearrangement-independent fusions

Mechanisms leading to RNA-level fusion synthesis include *trans*-splicing, transcriptional readthrough and *cis*-splicing between adjacent genes (*cis*-SAGe) (Figure 1B). Fusion RNAs arising from splicing mechanisms and transcriptional readthrough are characterized by significantly closer breakpoints than those from genomic rearrangements [13].

Antisense-containing fusion transcripts add another layer of complexity to the fusion landscape. Sense–antisense (SAS) fusions, also known as cross-strand chimeras or intragenic *trans*-splicing events (Figure 1B), arise from the fusion of bidirectional transcripts of the same gene [16]. SAS fusions are typically tissue-specific, although many have been found recurrently across different tissue types [16, 17]. Mukherjee and Frenkel-Morgenstern found that most SAS fusions were annotated as long non-coding RNAs (lncRNAs) and could be involved in regulating gene expression, given that SAS fusions often interact with their parental mRNAs [7]. Furthermore, palindromic sequences are often overlapping the junction sites of SAS fusions, where they could generate a hairpin-like structure and lead to the formation of double-stranded RNA [17]. Approximately one fifth of SAS fusions contain palindromic sequences around the junction site rather than overlapping with it, but the distance from the breakpoint junction is significantly shorter than in non-SAS fusions [17].

### Fusion circRNAs

Linear chimeric RNA products are often the focus of fusion studies; however, circular fusion RNAs (fusion-circRNAs) are known as a possible outcome of back-splicing along the breakpoint of a gene fusion [18]. While circRNAs are recognized as important players in cancer and other diseases [19, 20], their subclass of fusion-circRNAs remains relatively unexplored. Known fusion-circRNAs include F-circSR, which originates from the SLC34A2-ROS1 fusion gene [21], and F-circEA-2a, which is derived from the *EML4-ALK* fusion gene [22]. Both fusion-circRNAs were found to promote cell migration in non-small cell lung cancer. Additionally, several studies have shown a gene fusion can encode for more than one kind of fusion-circRNA. By example, circBA9.3 [23] and F-circBA1 [24] promote cell proliferation while circBA1 inhibits cell proliferation [25], each derived from the *BCR-ABL1* gene fusion in chronic myeloid leukaemia (CML).

Other fusion-circRNA biogenesis mechanisms have been postulated, such as *trans*-splicing or read-through transcripts [26]. Most medulloblastoma samples express both the ARL17A-KANSL1 and the KANSL1-ARL17A fusion-circRNAs. The latter is likely generated via *trans*-splicing, given the KANSL1 gene is encoded downstream of ARL17A on chromosome 17 [26]. Of note, in a subset of medulloblastoma samples, both linear and circular KANSL1-ARL17A fusions were observed [26]. However, this study had no complementary DNA analysis to fully validate a genomic rearrangement-independent origin, nor was there any functional analysis to identify their role in cancer.

## The importance of RNA-level fusions

### Cis-SAGes in cancer

There is a debate on what are considered true fusion RNAs. Yuan *et al*. [27] proposed that only RNAs formed via *trans*-splicing or genomic rearrangement can be regarded as authentic fusions. This is because RNAs transcribed via a read-through mechanism could be RNAs of unannotated genes or RNA isoforms of known genes and therefore are not true fusions [27]. However, fusion RNAs may be functional gene precursors used to test functionality before fusion-encoding sequences are included in the genome [28]. A study found that *MRPS31P5* is not a pseudogene of *MRPS31* but a functional descendent of the *HNRNPA1L2-SUGT1* fusion RNA [28].

Other recent findings suggest that *cis*-SAGes can be functional in cancer and therefore should be considered in fusion detection studies. One example is the well-studied *cis*-SAGe fusion between solute carrier family 45 member 3 (*SLC45A3*) and the ETS transcription factor *ELK4* found in prostate cancer [29]. *SLC45A3-ELK4* translates to the same protein as *ELK4*; however, the abundance of fusion RNA is less than 1% of wild-type *ELK4* and regulates cancer cell proliferation as a lncRNA. When *SLC45A3-ELK4*, but not *ELK4* is silenced, the proliferation of prostate cancer cells is inhibited [29].

Two isoforms of the *cis*-SAGe *LHX6-NDUFA8* were detected exclusively in cervical cancer tissues and Pap smears and not in normal controls [30]. Additionally, *LHX6-NDUFA8* was more recurrent than other previously reported fusions in cervical cancer. While no significant correlation with clinical parameters was observed, Wu *et al*. [30] suggest that *LHX6-NDUFA8* expression may be an early event in cervical cancer tumorigenesis. This study further stimulated attention by identifying an additional recurrent *cis*-SAGe that is not found in normal tissue, *SLC2A11-MIF*. The silencing of *SLC2A11-MIF* resulted in cell cycle arrest and reduced cellular proliferation. This effect was unique to the fusion and not shared by the separate parental genes [30].

Another *cis*-SAGe with different properties from its parental genes is *RRM2-C2orf48*, which promotes cellular proliferation in colon cancer cells [31]. *RRM2-C2orf48* correlates with poor clinical outcomes; however, the expression of *RRM2* or *C2orf48* is associated with positive clinical outcomes. A similar pattern was observed for *BCL2L2-PABPN1* and *CHFR-GOLGA3*, which are upregulated in bladder cancer [32]. The two *cis*-SAGes were detected mostly in the nucleus, indicating they may act as lncRNAs.

Overall, these studies highlight the importance of *cis*-SAGes in the fusion landscape, as they may play a more important role in disease and cancer than previously thought. These studies emphasize the importance of using RNA sequencing (RNA-seq) instead of, or in addition to, DNA sequencing (DNA-seq) methods for fusion detection, as fusion events that occur at the RNA-level may be just as significant as genomic fusion events.

### RNA-mediated gene fusions

**Figure 2:**
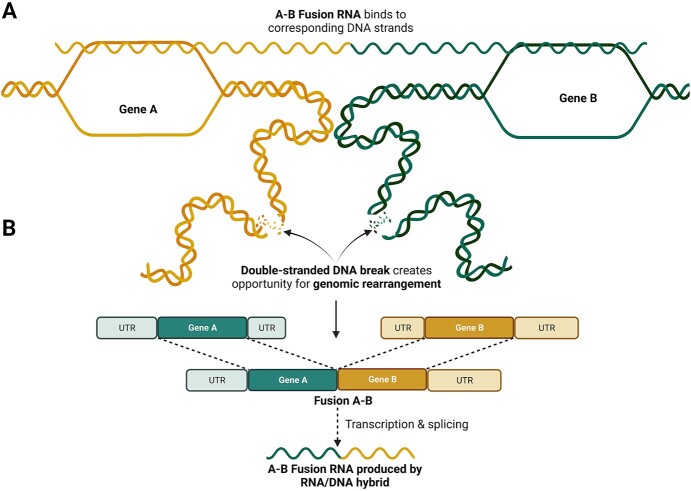
Theoretical model of RNA-mediated gene fusions. Genes A and B on separate chromosomes are within spatial proximity of each other. Subsequently, the A-B fusion RNA is produced via *trans*-splicing. (**A**) The A-B fusion RNA binds to chromosomal DNA of gene A and gene B in a sequence-dependent manner. (**B**) DNA breaks open the opportunity for genomic rearrangement guided by the RNA/DNA hybrid, which produces the final gene fusion A-B. UTR, untranslated region; created with BioRender.com

Presumably, gene fusions form before fusion RNA expression; however, studies have reported the detection of fusion RNAs independent of chromosomal translocations [6, 33, 34]. Transcriptomics and matched whole-genome sequencing data from tumours of 1188 individuals of the Pan-Cancer Analysis of Whole Genomes Consortium revealed that 18% of fusions displayed no evidence of genomic rearrangement [13]. Similar observations have spurred ‘the cart before the horse’ hypothesis, whereby fusion RNAs are initially produced by *trans*-splicing and recognize their parental genes (Figure 2A) and then possibly guide genome rearrangement to form the corresponding gene fusion (Figure 2B) [35]. Therefore, fusion RNAs could offer a unique opportunity to identify individuals at risk for genomic rearrangement and initiate intervention before a genomic oncogenic event occurs.

Experimental evidence in prostate and endometrial cells demonstrated that induced expression of *TMPRSS2–ERG* and *JAZF1-SUZ12* fusion RNA could facilitate genomic rearrangements [36, 37]. In prostate cancer cells, this process may be facilitated in part by physiological levels of testosterone, which alters chromosomal DNA looping and thereby moves the parental genes of the fusion into physical proximity. In endometrial cells, oestrogen and progesterone have the opposite effect leading to suppression of this event [36, 37].

Active gene transcription may prevent the sense fusion RNA from binding to the antisense DNA strand, as antisense fusion RNAs were preferential for inducing gene fusion [36, 37]. Supporting this notion, RNA polymerase-II inhibition enabled gene fusion generation by sense fusion RNAs [36]. Interestingly, antisense RNAs can arise from unrelated genomic sources that resemble a fusion RNA antisense to both parental genes, such as endogenous RNA *AZI1* for the *TMPRSS2–ERG* fusion in prostate cancer cells [36].

Whether this mechanism occurs *in vivo* awaits further investigation. It should be noted that antisense-containing fusion transcripts appear to be a common event, with a recent bioinformatics study demonstrating that they contribute to 61% of recurrent fusion transcripts across 33 cancers [38]. However, the proportion of fusion transcripts which have both fusion partners belonging to the anti-sense strand was not specified in the study.

### Fusion RNAs may provoke secondary fusion events

Guo *et al*. [39] proposed a novel mechanism whereby fusion transcripts spawn secondary fusions resulting in densely connected fusion networks. The authors found that the RNA fusion landscape is more complex than mRNA–mRNA fusions, which make up only 30.2% of fusions in cancer [39]. The remaining 53.7 and 16.1% of fusion events were mRNA–lncRNA and lncRNA–lncRNA fusions, respectively [39].

LncRNA-involved fusions, specifically mRNA-enhancer RNA (eRNA) fusions, may represent a mechanism for generation of secondary fusions via eRNA-mediated long-range interactions. In these events, the protein-coding fusion partner incorrectly connects with the targeted genes of the eRNA partner, forming oncogenic fusion RNA–gene interactions. This model, developed from the RNA-poise mechanism [40], is supported by the large number of fusion hub genes that have multiple fusion partners in fusion RNA-gene interaction networks [39]. This excess in fusion partners in fusion RNA–gene interaction networks is possibly due to the large number of targets for individual eRNAs [39].

## Detecting fusion transcripts via RNA-Seq

The sensitivity and specificity of detecting fusions with RNA-seq are dependent on sequencing depth, read length and quality, as well as the bioinformatics approach and parameters used. Bioinformatics fusion callers search for reads that map to more than one gene and call fusions where a minimum number of split-reads support the fusion breakpoint and additional read pairs (in paired-end sequencing protocols) span the breakpoint across the fusion partners.

Wang *et al*. identified thousands of recurrent SAS fusions in short-read RNA-seq data from The Cancer Genome Atlas cohort [16]. However, such data are not ideal for the *de novo* identification of SAS fusions due to the short length and non-stranded libraries used. Additionally, the fragmentation process during library preparation makes it unlikely to recover full-length SAS species. Short-reads are adequate for gene expression profiling and the quantification of well-known gene fusion transcripts; however, they do not perform as well in the discovery of novel and unconventional fusion transcripts. This is because short-reads are less efficient in capturing complex genomic rearrangements, repeat-rich regions or full-length transcripts. Identifying these challenging molecules requires the use of complex computational analyses to infer full-length transcript sequences. Consequently, biologically important variants may be missed.

Challenges in short-read-based fusion discovery are well known, and many methods have been developed to circumvent these issues. Short-read fusion detection software follows two principal approaches: (i) mapping-first: to identify discordant reads suggestive of genomic rearrangements and (ii) assembly-first: assembling reads into longer transcript sequences to identify fusion transcripts [41]. While the mapping-first approach is more sensitive, assembly-first is better at recovering fusion isoforms [41].

One approach to overcome the read-length limitation of short-read sequencing is synthetic long-read (SLR) sequencing, which relies on compiling together short-reads that share the same barcode, and subsequently constructing a longer read [42]. SLR-seq has been successfully employed to identify large-scale isoform redistributions and several previously unknown fusion isoforms in benign colon mucosa, primary colon cancer and metastatic colon cancer [43]. SLR provides a means to obtain the benefits of long-read sequencing at short-read sequencing costs, with low error rates and higher throughput. However, the fundamental unit of SLR assembly is still a short-read, therefore limiting assembly contiguity, homogenous coverage and the detection of large repeat regions. Moreover, when compared directly to long-read sequencing data, SLR displayed much shorter read lengths [44, 45]. Furthermore, SLR library preparation requires polymerase chain reaction (PCR) amplification, introducing bias due to inefficiency in regions with a high GC content [45].

## Long-read RNA-Seq: new possibilities for fusion transcript discovery

Advances in long-read sequencing technologies, such as PacBio and Oxford Nanopore Technology (ONT), allow the generation of reads, which are tens of kilobases in length at a relatively low cost. Notwithstanding that long-read sequencing is more expensive than short-read sequencing, long-reads can yield more accurate fusion predictions as they can span the full length of transcripts and thus increase mapping accuracy and fusion identification. Long-reads can resolve complex multi-exon isoforms and identify large transcripts, without relying on statistical inference. Mitsuhashi *et al*. [46] were able to use this advantage for detecting the full-length of the *LTR–RBM26* fusion transcript and differentiating between its two splicing isoforms. An additional advantage of long-read transcriptome sequencing is the ability to identify double-hop and bridged fusions [15]. Compared to short-read data, long-read data provide higher precision, although sensitivity is lower [14,47].

Long-read sequencing overcomes some limitations of short-read technologies and presents unique opportunities for the investigation of fusion RNAs and related alternative splicing and structural rearrangement events. However, long-read sequencing also introduces new challenges. The biggest limitations of long-read sequencing platforms are lower throughput compared to short-read platforms and lower per-base accuracy. ONT’s first-generation sequencers produced long-reads with relatively high error rates (on average 14% per-read for both direct RNA and cDNA sequencing) [48]. As a result, several strategies for error correction were developed [49]. Some use unique molecular identifier-guided error correction [50,51]; however, computational tools, such as *isONcorrect*, have also shown to be effective [52]. Using *isONcorrect*, Sahlin and Medvedev [52] obtained a median accuracy of 98.9–99.6%. In recent years, ONT have released new chemistries (e.g. Q20+) and base-calling algorithms to improve the accuracy of raw reads to 99.6%. PacBio also developed approaches to achieve higher accuracy with High Fidelity (HiFi) reads [53–55]. HiFi reads are produced via circular consensus sequencing, whereby multiple passes of a single molecule are used to derive a consensus sequence. HiFi reads can provide base-level resolution with 99.8% single-molecule read accuracy [53–55]. However, HiFi reads are limited by the number of passes required to achieve the desired accuracy and the overall read length [54].

Due to the lower read depth of long-read sequencing methods, lowly expressed fusion transcripts are unlikely to be captured or there is insufficient depth to conclusively find base fusion predictions [56]. For example, when Seki *et al*. [57] sequenced LC2/ad lung cancer cells, many fusion calls had only one supporting read, with the cancer driver fusion *CCDC6-RET* having seven reads directly matching the junction of the fusion [57]. Despite this, direct sequencing of RNA with ONT bypasses the issue of artificial chimeric molecules generated during cDNA library preparation [58]. Theoretically, ONT sequencing can also produce false-positive fusion events, as base calling software could have two molecules represented by a single read [59]. One advantage of the ONT is that it can be applied to directly sequence native RNA molecules, allowing the identification of RNA modifications, such as methylation [60]. While RNA modifications have been described in the context of splicing regulation [61], their role in *trans*-splicing and fusion RNA synthesis is yet to be determined. Direct RNA-seq can also capture transcripts that may be missed during cDNA synthesis due to length or sequence complexity.

Few studies have used a long-read RNA-seq approach to detect fusion transcripts in cancers; however, the benefits have been recognized. Due to the initial high error rates associated with long-read sequencing technologies, earlier studies employed a hybrid sequencing approach to correct long-read errors with short-read data [62,63]. Weirather *et al*. [62] developed a fusion detection algorithm that integrates both short- and long-read sequencing data [62]. They applied IDP-fusion to PacBio and Illumina RNA-sequencing data from MCF-7 breast cancer cells and compared the tool to short-read-only approaches. IDP-fusion detected gene fusions with higher precision and lower false positive rate. Although the study utilized a hybrid-sequencing approach, the sensitivity of fusion detection by IDP-fusion had limited dependence on short-reads, and the precision was increased compared to PacBio’s Iso-Seq analysis pipeline. Long-reads can also be beneficial for unravelling the complexity of fusion isoforms and splicing events within tumorigenesis-related gene fusions. For example, eight expressed fusion isoforms of the well-known *BCAS4–BCAS3* fusion and three different break points were identified [62]. Cheng *et al*. [64] combined PacBio’s single-molecule real-time (SMRT) sequencing with Illumina RNA-seq to examine the transcriptomic landscape in oesophageal squamous cells. They identified 1972 transcript fusions from full-length SMRT reads, which were enriched for genes related to RNA processing and cancer signalling pathways. Despite the multitude of fusion transcripts identified, few were found in the ChimerDB database (kobic.re.kr/chimerdb), suggesting that most fusions detected with long-reads were novel. A comparison of SMRT and Illumina assembly indicated that SMRT sequencing identified 5–10 times more fusion transcripts [64].

Longer reads have also been utilized to identify complex fusions, such as multi-segmented fusions. Namba *et al*. [15] developed a computational pipeline for Multi-Sample long-read Transcriptome Assembly to construct a cohort-wide transcriptome from SMRT sequencing data of 22 breast cancer patients [15]. The double-hop fusions identified had intergenic regions of thousands of base pairs, and some fusions displayed splicing in these regions. Nattestad *et al*. [14] also detected hopped fusions in HER2-positive breast cancer cells. *CPNE1-PREX1* had previously been discovered using RNA-seq data and was validated with PCR [65]. Long-reads in this study were able to capture the two genomic rearrangement events together in a single read [14]. Additionally, a novel ‘3-hop’ gene fusion between *KLHDC2* and *SNTB1* with sequences from three different chromosomes was observed [14]. This fusion event was previously misreported as a product of two genomic rearrangements [66]; however, both 2-hop and 3-hop paths result in the same gene fusion [14]. It should be noted that genomic variants are not direct indicators of mature RNA and *vice versa*. For example, Hu *et al*. [67] detected a three-segment fusion involving the *RECQL5* gene and two segments from chromosomes 8 and 7 using long-read DNA-seq in breast cancer samples. However, paired long-read RNA-seq revealed that the chromosome 7 fragment was removed through splicing resulting in a mature transcript with an overall shorter length [67].

In sum, long-read sequencing has proven to be a promising methodology for discovering novel isoforms, fusion and splicing events, which would otherwise not be detected with short-reads.

## Available fusion assays

RNA-seq has facilitated genetically guided treatments and patient management, utilizing small biopsies more efficiently and investigating the mutational status of multiple genes in a single assay. A range of targeted short-read RNA-seq fusion detection assays has been developed [68–70], and several are commercially available, including amplicon-based (FusionPlex, AmpliSeq, QIAseq, RNAscan, Oncomine Focus Assay) and hybrid capture-based (TruSight Tumor 170, SureSelect XT) assays [71,72]. However, relatively few long-read-based RNA-seq fusion detection assays have been developed thus far.

Of notable interest, Cavelier *et al*. [73] developed a targeted sequencing assay to directly detect the entire 1578 bp *BCR-ABL1* fusion transcript on PacBio’s RSII system. Complementary DNA (cDNA) from six CML patients with poor response to tyrosine kinase inhibitor therapy was analysed. The custom assay was able to identify all previously identified mutations, in addition to several additional mutations that were undetected by routine clinical diagnostic analysis. Furthermore, this assay had a relatively quick turnaround of 2–3 days.

Jeck *et al*. [74] developed an amplicon-based ONT sequencing assay for the same-day detection of fusion transcripts in acute myeloid leukaemia (AML). All analyses were completed in 13 h, and most of the results were available after 2 h of sequencing. The assay uses an anchored multiplexed PCR method, modified from Illumina libraries. The assay covers 52 genes associated with AML and enabled the detection of 16 fusions in 11 of 16 samples.

Following the release of ONT’s flow cell dongle (a.k.a. Flongle), Jeck *et al*. [75] sought to retest 15 AML specimens. While Flongle flow cells (126 channels) provide lower throughput than MinION flow cells (512 channels), Flongle flow cells may offer lower price testing. All fusions previously identified with MinION flow cells were also recovered with the smaller Flongle flow cells, with the extra positive identification of a *PML-RARA* fusion previously missed due to inadequate coverage depth. Furthermore, this assay was able to detect *CIC-DUX4* translocations that were not initially detected by the Illumina sequencing pipeline. *DUX4 t*ranslocations are challenging to detect with short-read sequencing because of repetitiveness and complexity of the locus. Although the Flongle sequencing pipeline reduced the sequencing time, the library preparation takes >16 h with the Flongle system.

## Bioinformatic approaches for fusion detection in long-read data

Several fusion detection tools have been developed to detect fusions in short-read RNA-seq data [76–78]. These tools are specific for short-read RNA-seq because they correct for biases inherent to short-read sequencing protocols and ambiguous alignment to different transcript isoforms. Recently, several fusion callers have been developed to handle long-read-only data from either direct RNA or cDNA sequencing from both PacBio and ONT platforms (Table 1).

**Table 1:** Long-read fusion detection software tools and features

Name	Aligner	Reference alignment	Fusion classification	Read-through filter	Transcript quantification	Call quality	Ref
**RNA-seq**							
LongGF	Minimap2 [79] splice aware mapper	Genome	No	No	No	Requires manual validation (IGV), can adjust number of supporting reads filter	[80]
AERON	GraphAligner [81] sequence-to-graph	Transcriptome	No	No	Yes	Requires manual validation, some statistical validation	[82]
Genion	desalt [83] splice aware mapper	Genome	Gene fusion Read-through *Trans*-splicing either merged with gene-fusion or removed due to low-expression.	<500 kbp	No	Stringent statistical filtering	[59]
JAFFAL	Minimap2 [79] splice aware mapper	Transcriptome and Genome	Gene fusion *Trans*-Splicing Read-through filtered out, recoverable	<200 kbp	No	Some statistical filtering	[58]
**DNA-seq**							
NanoFG	Minimap2 [79] splice aware mapper	Genome	Gene fusion only	N/A	N/A	Minimum two fusion-supporting reads	[84]
**Fusion-circRNA**							
circfull	Minimap2 [79] splice aware mapper	Genome	No	No	Yes	Requires manual validation (IGV), can check number of supporting reads	[85]

One of the first long-read fusion callers developed for transcriptomic data was LongGF. LongGF takes a BAM file and a GTF file as input, the latter containing the definition of known genes and transcriptional isoforms, and outputs a prioritized list of candidate gene fusions with supporting long-reads [80]. LongGF filters out overlapping genes, overlapping alignments and distant alignments of reads before clustering the reads together. Additionally, users can define a threshold for the overlap between mapped genomic positions in an alignment and exons to be considered a transcript. There are several limitations associated with LongGF. Mapped genomic positions are compared to pre-defined genomic coordinates of exons for each genic transcript. Therefore, fusions involving unknown genes and exons cannot be detected. LongGF may also experience issues with homologous genes, as the sequence similarity can complicate assignment of where a fusion partner originates. Moreover, LongGF may miss gene fusions containing very short genes because alignments must be greater than 100 bp and have significant overlap with a certain gene to be included for further analysis. While users can set a lower threshold to capture smaller segments, this may consequently risk introducing false positives.

Genion was developed with more stringent thresholds in an attempt to minimize false positives with statistical testing [59]. Unlike LongGF, which applies a series of filters to individual reads before clustering them, Genion applies filters to whole read clusters. This approach provides greater filtering power and cleaner information to analyse filtered candidates. However, this increases total computation time [59]. Both LongGF and Genion filter candidates based on the overlaps of genes and alignments. Genion, however, does not filter reads with long distance between alignments to avoid mapping errors and genomic variants [59]. Another advantage of Genion is the retainment of *cis-*SAGE results. However, *trans*-splicing fusions may not be distinguishable from lowly expressed gene fusions. Despite the improvements from LongGF’s oversensitivity, Genion may be at risk of having too stringent filtering. By example, analysis of a MCF-7 dataset revealed that several validated fusions were filtered out due to lower read support or because gene partners were overlapping [59].

Rautiainen *et al*. [82] developed AERON, which is the first long-read fusion detection tool to use a sequence-to-graph alignment tool, known as GraphAligner [81]. AERON also quantifies the transcripts by counting the number of reads assigned to it and converting the count into Transcripts Per Million values. However, as is typical when attempting to quantify transcripts in long-read data, there is limited correlation with short-read data because of differences in sequencing depth. AERON, unlike LongGF and Genion, aligns the reads to a reference transcriptome, rather than a reference genome. One limitation associated with AERON is that the assignment of reads to transcripts is affected by highly similar and short-length transcripts. Rautiainen *et al*. [82] noted the accuracy of the fusion candidates is dependent on each gene partner having at least 700 bp reliably detected. However, this length-dependent limitation was related to the higher error-rate associated with long-read sequencing, which is likely to have been improved with current technologies.

Recently, Davidson *et al*. [58] released JAFFAL, developed from the short-read fusion caller JAFFA. JAFFAL is distinguished from other long-read fusion callers as the reads undergo two rounds of alignment via Minimap2. The reads are first aligned to a reference transcriptome. Reads identified to have sections aligning to different genes in the first alignment are then aligned to a reference genome. This double alignment is meant to minimize false positives and reduce computational time as only a small subset of reads is aligned to the reference genome. An additional advantage is JAFFAL’s ability to filter out cDNA chimeras. Because splice sites are often preserved, fusion transcripts are likely to display the breakpoint in RNA at the start or end of an exon. JAFFAL attempts to prioritize breakpoints and classify them based on number of supporting reads and whether or not the breakpoints align to exon boundaries. Where an event is supported by a single read with breakpoints aligning to exon boundaries, JAFFAL may falsely annotate this event as ‘Potential Trans-Splicing’. JAFFAL also filters out *cis*-SAGe events, identified by breakpoints within 200 kbp of each other, and fusions which involve the mitochondrial chromosome. However, these events can be recovered by the user. Another unique feature of JAFFAL is the ability to detect multi-segmented fusion events, by searching for reads with two or more breakpoints. Like LongGF, there is a dependence on annotated transcripts. Fusions with breakpoints in intergenic or intronic sequences are not detected; however, this limitation is shared by most fusion callers.

DNA-seq data analysis can be used for supplementary validation of gene fusion events detected with transcriptomic approaches. NanoFG [84] is the only published tool that identifies gene fusions in long-read DNA-seq data generated from ONT devices. Like several aforementioned tools, NanoFG aligns reads to a reference genome using Minimap2 [79]. However, NanoFG has some flexibility with its pipeline, allowing users to extract structural variants (SVs) with either NanoSV [86] or Sniffles [87]. NanoFG then selects potential gene fusion events from SVs with both ends annotated with genes from the ENSEMBL database. NanoFG subsequently remaps all candidate fusions using LAST [88] and defines the breakpoints with NanoSV before selection for fusions likely to produce a continuous transcript on the same strand. Like JAFFAL, NanoFG optionally allows users to identify complex fusion events where multiple breakpoints occur on the same read. However, NanoFG does this by classifying the first and last break-end in the read as an additional SV, because smaller SVs near the fusion breakpoint inhibit default NanoFG from detecting the fusion.

While several tools have been developed to detect circRNAs in RNA-seq data [89], very few have been developed for long-read sequencing data. Currently published tools for identification of circRNAs from long-read sequencing data include CIRI-long [90], isoCirc [91] and circfull [85]. Of these only circfull has been designed with fusion-circRNAs in mind. circfull was developed for the analysis of circFL-seq data, a full-length circRNA sequencing method on ONT platforms. circfull maps reads to a reference genome with minimap2. Aligned reads with chiastic overlapping segments are considered candidate circRNA reads and subsequently labelled a normal, fusion on different chromosomes and fusion on the same chromosome (when mapping to two separate loci >1Mbp apart). Therefore, fusion-circRNAs originating from a readthrough mechanism <1 Mbp are not immediately classified as fusion-circRNAs but instead as normal circRNAs. The boundaries of the chiastic segment of the candidate circRNAs are considered as potential back-splicing junctions while forward-splicing junctions are determined by the skipped region from the reference. Normal circRNAs are considered to have one fusion junction from back-splicing, and fusion-circRNAs are considered to have two fusion junctions with the additional junction coming from the gene fusion.

### Algorithmic ignorance of adjacent gene fusions

Many fusion callers, including those discussed above, filter out *cis*-SAGes and fusions resulting from *trans*-splicing. This is likely a result of design to identify gene fusions rather than transcript fusions, or because of the premise that these types of fusions are functionally insignificant. However, fusion RNAs that are present in healthy tissue could be differentially expressed in cancer and might have dose-dependent functionality. An example of this is the fusion of the cell surface receptor CLEC12A and the miRNA-223 host gene (*MIR223HG*), which is expressed in healthy cells but at higher levels in CML patients and in pro-monocytic cells resistant to chemotherapy [33]. *CLEC12A-MIR223HG* translates into a fusion protein with potentially altered functions as compared to wild-type *CLEC12A*.

Wu *et al*. [30] highlighted the issue of *cis*-SAGe fusion RNAs being wrongly clustered and thus missed by bioinformatics detection methods. *LHX6-NDUFA8* was originally grouped into a different category from *cis*-SAGe because the *MORN5* gene sits between *LHX6* and *NDUFA8*. However, *MORN5* is transcribed from the opposite strand, and the primary transcript connecting *LHX6* and *NDUFA8* was observed. By manual re-examination of 425 recurring fusion RNAs observed in cervical cancer, 37 new candidates for *cis*-SAGe fusions were identified. Given that recent studies have demonstrated *cis-*SAGes can play crucial roles in cancers, future programmatic approaches of fusion transcript detection should avoid filtering them out.

## Experimental validation and functional characterisation of fusion RNA

**Figure 3:**
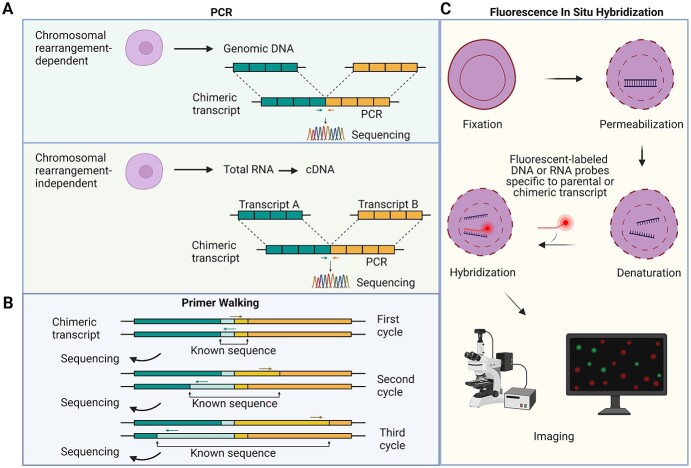
Overview of fusion validation strategies. Experimental validation of individual fusion transcript can be performed with either PCR-based or imaging-based techniques (FISH). (**A**) PCR amplification with primers flanking the predicted breakpoint followed by Sanger sequencing allows accurate fusion validation. (**B**) Primer walking can determine the full-length sequence of the fusion transcript complimented by long-read sequencing. This technique involves iterative rounds of PCR followed by Sanger sequencing allowing a ‘walk’ through an unknown sequence. (**C**) FISH, fluorescently labelled probes with a sequence complementary to the break point are synthesized. These probes are then hybridized to the target sequence followed by visualization of the fluorescent signal. As controls, probes complementary to parental transcripts need to be used. Created with BioRender.com

This section summarizes pipelines one could follow to dissect the biological relevance of a fusion RNA *in vitro*. The relevance depends both on a fusion RNA’s expression level and the biological functions of the parental genes. Some fusions might merely be a passenger (not a driver) of a biological phenomenon or disease. Therefore, vigilance is necessary to carefully plan and validate the fusion transcripts using rigorous, independent, complementary and stringent protocols. For the validation of a specific fusion transcript identified from RNA-seq data, FISH (fluorescence *in situ* hybridization) and PCR-based techniques are commonly used (Figure 3).

FISH is a technique, which allows the visualization of a target DNA (DNA-FISH) or RNA (RNA-FISH) complementary to an artificially designed fluorescently labelled probe. Utilizing DNA and RNA-FISH allows distinction between fusions transcripts that are genomic rearrangement-independent. RNA-FISH utilizes fluorescently labelled probes that hybridize with target transcripts and allows their visualization in cell cultures, tissues or whole-mount preparations [92, 93]. Although highly informative, this technique presents some limitations when studying fusion transcripts. Establishing an RNA-FISH protocol with unvalidated probes can be extremely time-consuming and several technical variables can affect signal detection. These factors include probe design and validation, target cell/tissue preparation and optimization of conditions for pre- and post-hybridization treatments. A single-molecule FISH with two differently coloured probes has been utilized for the detection of fusion transcripts. Each probe is specific to individual fusion partner. This enables the fusion transcripts to be observed as distinct spots that fluoresce in both colours [93].

In PCR-based detection of a fusion transcript, cDNA is first synthesized from the total RNA. DNase treatment prior to cDNA synthesis is essential. Next, primers specific to fusion breakpoints are designed and used in either quantitative or qualitative PCR reactions. PCR products can be sequenced to confirm the breakpoint. Furthermore, the primer walking technique can be used to determine the full-length of the fusion transcript [33]. Long-read RNA-seq data can complement primer walking to determine the full length of the fusion transcript. To distinguish fusions occurring at the RNA level from chromosomal translocations, PCR can be performed on the genomic DNA.

Due to the necessity of a polymerase-based extension step, transcripts generated by template switching (TS) can be misdiagnosed as novel isoforms [94]. TS refers to the ability of DNA polymerases to initiate transcription from a second strand while still being bound to the first one [95]. The TS phenomenon is well recognized and can give rise to artificial transcripts, which can be misinterpreted as novel fusion transcripts. Moreover, 35–55% of total fusion transcripts identified might be *in vitro* artefacts [95, 96]. Of 40 fusion transcripts chosen from four well-established databases, only 13 could be validated with RT-PCR using either of the two reverse transcriptase used. Interestingly, only 6 of these 13 transcripts were amplified with both reverse transcriptases [95]. Subsequent experiments were performed to test TS by mixing the total cellular RNA with a FLAG-tagged 3′-fusion partner followed by a FLAG-primed reverse transcription. In the absence of TS, a co-linear cDNA is expected, whereas a non-co-linear cDNA containing the 5′-fusion partner will be detected following TS. A high frequency of TS was reported for the seven false-positive fusion transcripts [95].

Some modifications in the cDNA synthesis step can reduce TS including an increase in the extension temperature during cDNA synthesis [97]. Moreover, the propensity of a given transcript to accommodate TS should be studied and validation should also be performed by directly studying the fusion RNA whenever possible using techniques like RNA-FISH. Additionally, quality controls need to be applied to the RNA-seq data specifically designed to eliminate false-positive detection of fusion transcripts [98, 99].

A fusion transcript can either function as a lncRNA or produce a novel protein [29, 100]. The nature of the dataset or parental genes can inform whether a fusion transcript has a function in a biological context. To functionally characterize a fusion transcript, its expression level can be modulated by overexpression or knockdown approaches, and phenotypic changes in the cells can be monitored [31]. Overexpression of a fusion transcript can be achieved with traditional viral or non-viral vector-based technologies while small interfering RNAs specific to the fusion transcript have been widely used in knockdown studies [16, 29, 30]. For instance, siRNA-mediated loss of function of the *SCLC45A3-ELK4* fusion but not of the parental *ELK4* transcript resulted in reduced growth of prostate cancer cells. The phenotype could be rescued by retrovirus-mediated overexpression of the fusion transcript [29, 101]. In another study, lentivirus-mediated overexpression of the *NPM1-TYK2* fusion provided evidence that it is a driver of lymphoid cell transformation. Further confirmation with cell viability and clonogenicity assays was complemented by the observation that *NPM1-TYK2* overexpression is tumorigenic *in vivo* [3]. Similarly, siRNA-mediated knockdown of the *SCL12A11-MIF* fusion transcript but not the transcripts of the parental genes resulted in a reduction of cervical cancer cell proliferation. To further confirm the role of the fusion transcript, rescue experiments were performed with lentivirus-mediated overexpression [30].

The changes in transcriptomic landscape and pathways affected can provide further insights into the role of the fusion transcript [30]. Cell-based assays including proliferation, apoptosis, cytotoxicity, senescence and viability assays can be performed, with or without a therapeutic intervention. Finally, *in vivo* tumorigenic properties of a particular fusion transcript can be studied by subcutaneous tumour experiments by implanting cells which overexpress or underexpress the fusion transcript.

## Conclusion and perspective

Gene fusion events in cancers are of great importance as they can determine clinical management and outcome. However, the dominant interest in DNA-level fusion events has caused RNA-level fusion events and their role in cancer and disease to be generally overlooked. Yet, growing evidence suggests that RNA-level fusions can be involved in multiple cancer-related processes or can provide novel biomarkers for diagnosis and prognosis.

Debate surrounds the classification of fusion transcripts, particularly *cis*-SAGes as they may potentially be RNAs of unannotated genes or variants, which are present in healthy cells. More investigation is needed to characterize fusion transcripts in normal biology, as disease states may also arise from changed expression or localization rather than their simple presence or absence. Furthermore, fusion transcripts putatively facilitate gene and secondary RNA-level fusion events. Therefore, fusion transcripts serve as potential biomarkers and targets for prevention of carcinogenic gene fusions.

Current bioinformatic tools for fusion detection from long-read sequencing data are few, and each has its own unique parameters and assumptions. However, all were designed to detect gene fusion events rather than fusion transcripts. They were designed to filter out *cis-*SAGes and *trans-*splicing events. Given the roles RNA-level fusions may have in cancer and other diseases, future fusion detection software should be inclusive of these fusion events.

The advent of long-read sequencing technology has made it possible to characterize more complex fusion events and capture the entire length of the transcript, leading to less reliance on statistical inference for alignment and more accurate fusion calls. Recent studies have employed long-read sequencing for fusion detection, thereby offering a promising methodology for discovering novel fusion events, isoforms and splicing events, which would otherwise not be detected with short-reads. Improvements in sequencing technology with associated reductions in error rates should encourage further refinement of fusion detection assays and software for long-read sequencing to benefit human health.

**Key Points**

- Fusion transcripts can be synthesized via both genomic rearrangement-dependent and -independent mechanisms.
- RNA-level fusions are often overlooked due to their presence in normal biology; however, recent studies support a functional role in cancer.
- Because of bias towards searching for gene fusion events, most bioinformatic tools have built-in parameters to exclude RNA-level fusions.
- Long-read sequencing provides new opportunities in fusion detection, particularly with regard to complex fusion events and elucidating splicing isoforms.
